# Nutritional and metabolic status of children with autism vs. neurotypical children, and the association with autism severity

**DOI:** 10.1186/1743-7075-8-34

**Published:** 2011-06-08

**Authors:** James B Adams, Tapan Audhya, Sharon McDonough-Means, Robert A Rubin, David Quig, Elizabeth Geis, Eva Gehn, Melissa Loresto, Jessica Mitchell, Sharon Atwood, Suzanne Barnhouse, Wondra Lee

**Affiliations:** 1 Arizona State University, Tempe, AZ, USA; 2 Health Diagnostics, South Amboy, NJ, USA; 3 Integrative Developmental Pediatrics, Tucson AZ, USA; 4 Department of Mathematics, Whittier College, Whittier, CA, USA; 5 Doctor's Data, St. Charles, IL, USA; 6 Southwest College of Naturopathic Medicine, Tempe, AZ, USA

## Abstract

**Background:**

The relationship between relative metabolic disturbances and developmental disorders is an emerging research focus. This study compares the nutritional and metabolic status of children with autism with that of neurotypical children and investigates the possible association of autism severity with biomarkers.

**Method:**

Participants were children ages 5-16 years in Arizona with Autistic Spectrum Disorder (n = 55) compared with non-sibling, neurotypical controls (n = 44) of similar age, gender and geographical distribution. Neither group had taken any vitamin/mineral supplements in the two months prior to sample collection. Autism severity was assessed using the Pervasive Development Disorder Behavior Inventory (PDD-BI), Autism Treatment Evaluation Checklist (ATEC), and Severity of Autism Scale (SAS). Study measurements included: vitamins, biomarkers of vitamin status, minerals, plasma amino acids, plasma glutathione, and biomarkers of oxidative stress, methylation, sulfation and energy production.

**Results:**

Biomarkers of children with autism compared to those of controls using a t-test or Wilcoxon test found the following statistically significant differences (p < 0.001): Low levels of biotin, plasma glutathione, RBC SAM, plasma uridine, plasma ATP, RBC NADH, RBC NADPH, plasma sulfate (free and total), and plasma tryptophan; also high levels of oxidative stress markers and plasma glutamate. Levels of biomarkers for the neurotypical controls were in good agreement with accessed published reference ranges. In the Autism group, mean levels of vitamins, minerals, and most amino acids commonly measured in clinical care were within published reference ranges.

A stepwise, multiple linear regression analysis demonstrated significant associations between several groups of biomarkers with all three autism severity scales, including vitamins (adjusted R^2 ^of 0.25-0.57), minerals (adj. R^2 ^of 0.22-0.38), and plasma amino acids (adj. R^2 ^of 0.22-0.39).

**Conclusion:**

The autism group had many statistically significant differences in their nutritional and metabolic status, including biomarkers indicative of vitamin insufficiency, increased oxidative stress, reduced capacity for energy transport, sulfation and detoxification. Several of the biomarker groups were significantly associated with variations in the severity of autism. These nutritional and metabolic differences are generally in agreement with other published results and are likely amenable to nutritional supplementation. Research investigating treatment and its relationship to the co-morbidities and etiology of autism is warranted.

## Background and Significance

Vitamins, minerals, and essential amino acids are, by definition, essential for human health, primarily due to their critical function as enzymatic cofactors for numerous reactions in the body, such as the production of neurotransmitters and fatty acid metabolism Historically attention has focused on inadequate intake of vitamins and minerals due to poor diet as a major contributing factor to many child health problems in the US and around the world, including anemia (low iron), hypothyroid (low iodine), scurvy (vitamin C deficiency), and rickets (calcium and/or vitamin D deficiency). However, nutritional status depends not only on intake, but also on digestion, absorption, metabolic processing, and metabolic demand. More recently the focus has shifted to the relationship between relative metabolic disturbances and developmental disorders, for example those associated with Attention Deficit Disorder [1-5], learning disorders [6], and intellectual development [7]. We hypothesize that nutritional insufficiency and metabolic imbalances may play a role in autism spectrum disorders (ASD).

There have been several studies of the nutritional and metabolic status of children with autism, but each focused on study of only a few biomarkers. Three studies have demonstrated that children with autism have impaired methylation, decreased glutathione, and oxidative stress [8-10], and those studies demonstrated that nutritional supplementation (with vitamin methyl-B12, folinic acid, and trimethylglycine) is beneficial. One study in Romania found normal levels of vitamin B12 and folate in children with autism compared to controls, but low levels of plasma glutathione [11]. Several other studies have also demonstrated increased oxidative stress [12-15]. One study [16] found that children with autism had high levels of plasma vitamin B6 pre-supplementation, and this finding was confirmed in a follow-up study [17], suggesting a metabolic imbalance in B6. One study of dietary intake of 111 autistic children in China found that most had inadequate intake of folic acid, vitamin B6, vitamin A, Vitamin C, and zinc [18]. One study of vitamin D status in Egypt found that young children with autism had lower levels of vitamin D, both 25(OH)D and 1,25(OH)(2)D compared to age-matched controls [19]. One study in Slovakia found that children with autism had significantly higher levels of vitamin C and beta-carotene, but normal levels of vitamin A and vitamin E, compared to older teen controls [20].

There are several studies of minerals in children with autism. One study found that young US children with autism and their mothers had unusually low levels of lithium compared to neurotypical children and their mothers; lithium is receiving increasing recognition as being an essential mineral [21]. Two large studies of iron status found that US and Canadian children with autism had anemia in 8% and 16% of cases, respectively [22,23]. One small study of minerals in red blood cells found that young Canadian children with autism (n = 20) had lower levels of RBC selenium and RBC molybdenum than neurotypical children (n = 15) of the same age [24], but similar levels of most other minerals. A small study of zinc and copper in plasma found that British children with autism (n = 20) had similar levels to neurotypical children (n = 30) [25]. In contrast, a study of Turkish children with autism (n = 45) found that they had lower levels of zinc in plasma and RBC compared to neurotypical children (n = 41) [26]. One study [27] reported low levels of plasma zinc and high levels of serum copper in young children with autism as compared to published reference ranges, but.the lack of in-study controls is a weakness of this study.

There have been several studies of essential amino acids in autism with conflicting results. Increased levels were found by Aldred et al 2003 [28], both increased and decreased levels by Moreno et al 1996 [29], and decreased levels by Rolf et al 1993 [30] and Arnold et al 2003 [31]; the latter found only decreased methionine in the autism group on a standard diet. One limitation of the studies was their small population size (less than 25 participants in each arm). Another very important limitation is that fasting status was unclear in two of the studies [29,30] or only involved a limited (2-4 hours) fast in another study [31]. Only one of the studies [28] involved overnight fasting; this is important as amino acid values are not comparable unless all are done in a fasting state. One of the studies [28] involved very different age ranges for the controls and the autistic group which is important as pediatric reference ranges for some plasma amino acids vary substantially with age [32]. Thus, larger, more rigorous studies are needed.

The purpose of this study is to investigate the nutritional and metabolic status of children with autism compared to neurotypical children of similar age and gender, and to determine if some nutritional and metabolic biomarkers may be associated with the severity of autism. This study includes a broad array of biomarkers because that helps provide a more complete understanding of nutritional status, including vitamins, minerals, amino acids, and other metabolic biomarkers. The children with autism who participated in this study then continued into a randomized, double-blind, placebo-controlled study of the effect of a vitamin/mineral supplement, and the details of that follow-on investigation are reported in two companion papers [Adams et al, Effect of a Vitamin/Mineral Supplement on Children with Autism: Part A Nutritional and Metabolic Results, submitted, and Adams et al, Effect of a Vitamin/Mineral Supplement on Children with Autism: Part B. Effect on Symptoms.]. A strength of this study is the use of neurotypical controls of similar age, gender and geographic distribution, tested concurrently under identical conditions to the autism group, with blinded evaluation of samples by the laboratories.

### Methodology

This paper reports on the baseline levels of children with autism compared to neurotypical children. Neither group of children had taken any vitamin/mineral supplements in the two months prior to the study. This study was conducted with the approval of the Human Subjects Institutional Review Board of Arizona State University, study protocol number 0801002499.

#### Participants

Participants were recruited during May to December 2008 from Arizona with the help of the Autism Society of Greater Phoenix and the Arizona Division of Developmental Disabilities. All parents and children, where appropriate for age and developmental ability, signed parent consent/child assent forms.

### Enrollment criteria

1) age 5-16 years old;

2) no usage of a vitamin/mineral supplement in the last 2 months

3) no current use of any chelation treatment

4) Autism Group: prior diagnosis of autism, PDD/NOS, or Asperger's by a psychiatrist or similar professional, with written verification (no additional assessment was done in this study)

5) Control Group: in good mental and physical health, and no siblings with autism spectrum disorders, and no evidence of Attention Deficit Disorder by parent report (no additional assessment was done in this study)

### Participants

The characteristics of the study participants are listed in Table 1, and their physical and behavioral symptoms (per the ATEC) are listed in Table 2.

**Table 1:** Characteristics of Participants

	Autism Group	Neurotypical Group
**Total Participants**	55	44

**Male**	49 (89%)	39 (89%)

**Female**	6 (11%)	5(11%)

**Age (years)**	10.0 +/- 3.1	11.0 +/- 3.1

**Diagnosis**	85% autism, 4% PDD/NOS, 11% Asperger's	In good mental and physical health

**Medications**	55% no medications 29% psycho-pharmaceuticals - primarily risperidone and clonidine; 9% on CNS stimulants (primarily Concerta); 4% on anti-convulsants 5% on GI medications; 7% on asthma/allergy medicine; 2% on insulin	89% no medications; 9% on anti-inflammatories (asthma/allergies); 2% on anti-incontinence medication

**Special Diets**	84% on regular diet; 3 gluten-free, casein-free diet; 1 gluten-free; 3 reduced dairy/gluten; 2 low sugar	1 child on dairy-free diet

**Nutritional Supplements**	1 on fish oil; 2 on melatonin	none

**PDD-BI (modified autism composite)**	-63 +/- 54	n/a

**ATEC**	64 +/- 25	n/a

**SAS**	4.9 +/- 2.4	n/a

**Table 2:** Symptoms of Autism Participants, per the ATEC Subscale on Health/Physical Behavior

Symptom	% with moderate or severe problem
bedwetting	20%

wets pants/diapers	16%

soils pants/diapers	20%

diarrhea	18%

constipation	41%

sleep problems	40%

eats too much/little	68%

limited diet	53%

hyperactive	40%

lethargic	17%

hits/injures self	18%

hits/injures others	24%

destructive	24%

sound sensitive	44%

anxious/fearful	27%

unhappy/crying	14%

seizures	4%

obsessive speech	33%

rigid routines	35%

shouts/screams	50%

demands sameness	43%

often agitated	41%

not sensitive to pain	30%

hooked or fixated on certain objects	63%

repetitive movements	38%

This section was rated on a scale of 0 (none), 1 (mild), 2 (moderate), 3 (severe). Below are listed the percentages with moderate or severe problems, as reported by parents.

### Study Protocol

1) Participant parents contacted the study coordinator, and the study was explained by telephone. Consent/assent forms were sent to the parents for review, and then signed copies were brought to the study coordinator. The Principal Investigator (J.B. Adams) also discussed the study personally with each participant.

2) Parents of children with autism completed three questionnaires relating to the severity and symptoms of autism (see below).

3) The study physician conducted a physical exam to determine that the children were in adequate health for participating in the study.

4) Morning blood samples (50 ml) were collected after an overnight fast (8-12 hours). Morning urine samples were collected, and in almost all cases these were first-morning (overnight) urines.

5) All study data (questionnaires and laboratory samples) were assigned a coordinating subject code. All laboratory analyses were done blinded to subject group (Autism or Control).

#### Lab Measurements

Minerals and plasma amino acids were measured by Doctor's Data (St. Charles, IL, USA - http://www.doctorsdata.com). Vitamins, serum ferritin, and all other biomarkers were measured by Vitamin Diagnostics (South Amboy, NJ, USA; http://www.europeanlaboratory.nl). Both laboratories are certified by CLIA, the Clinical Laboratory Improvement Amendments program operated by the US Department of Health and Human Services which oversees approximately 200,000 laboratories in the US.

Measurement methods are summarized in Table 3. For urine analyses, correction for variations in dilution was done by adjusting for specific gravity [33] or by normalizing to grams of creatinine.

**Table 3:** Analytical methods for some of the measurements

Analyte	Source	Methodology
Vitamin A	Plasma	Spectrophotometry [80]

Total Carotenes (alpha, beta, epsilon, gamma)	Plasma	Spectrophotometry [81]

Vitamin C (sum of reduced and oxidized forms)	Plasma	Spectrophotometry [82]

Vitamin D3 (25-hydroxy)	plasma	Measured in plasma by liquid chromatography - tandem mass spectroscopy (LC/MS/MS) [83]

Vitamin E (total tocopherols, including alpha, gamma1, gamma2)	serum	Spectrophotometry [84]

Vitamin K	plasma	Vitamin K was extracted from plasma by methylene chloride in a monophasic design, purified on a C-18 cartridge, separated on a reversed-phase column, and then detected fluorometrically [85]

Thiamine	Whole Blood	Microbiological Assay [86]

Riboflavin	Whole Blood	Microbiological Assay [87]

Niacin	Whole Blood	Microbiological Assay [88]

Pantothenic Acid	Whole Blood	Microbiological Assay [89]

P5P	RBC	Microbiological Assay [90]

Biotin	Whole Blood	Microbiological Assay [91]

Folic Acid	Serum	Microbiological Assay [92]

Vitamin B12	Plasma	Microbiological Assay [93]

Choline (Free and Total)	RBC	Microbiological Assay [94]

Alpha lipoic acid	Plasma	Microbiological Assay [95]

CoQ10	Plasma	Reverse-phase high-performance liquid chromatography (HPLC) using hexane extraction on a C-18 column (15 cm, 5 micron) with methanol:hexane (95:5) as the mobile phase and UV detection [96]

Adenosine, Inosine, Uridine	Plasma	HPLC with 254 adsorption [97]

Formimino-glutaric acid (FIGLU)	Urine	Spectrophotometrically [98] after adjustment of specific gravity at neutral pH [99]

Kryptopyrole	Urine	Spectrophotometrically [100] after adjustment of specific gravity at neutral pH [99]

Methylmalonic Acid	Urine	Liquid chromatography - tandem mass spectroscopy (LC-MS/MS) with electrospray ionization [101], expressed per gram creatinine

N-methylnicotinamide	Urine	HPLC, with ultraviolet diode array detection [102], and the results are expressed per gram creatinine

Serum Ferritin	Serum	Immunometric assay with Immunlite 2000 (Diagnostics Product Corp., Los Angeles, California)

S-adenosylmethionine (SAM) and S-adenyosylhomocysteine (SAH)	RBC	Extracted from RBC [103] and measured by LCMS [104]

Glutathione (GSH and GSSG)	Plasma	Fluorescence detector [105]

Nitrotyrosine	Plasma	LCMS [106]

ATP	Plasma	Luciferin-luciferase assay [107]

NADH, NADPH	RBC	Spectrophotometry [108]

Sulfate (Free and Total)	Plasma	Using indirect atomic absorption spectrometry [109]

Vitamins were measured in the blood compartment (serum, plasma, or RBC) where they are most highly concentrated, or if evenly distributed intra- and extra-cellularly then whole blood was measured. Fat-soluble vitamins (A, D, E, K) are primarily concentrated in serum. For water-soluble vitamins, some are primarily in the plasma (like vitamin C), whereas others (like pantothenic acid) are significantly present in both serum and RBC, so whole blood was used. This approach then provides the best estimation of total body levels. Whole blood measurements are not commonly used for laboratory assessments because of challenges in processing the samples. However, by the use of vitamin-specific microbiological organisms as done in this study, whole blood levels are measured with a high degree of reliability.

Essential minerals were measured in RBC, serum, whole blood, and (for iodine) in urine. In most cases, serum reflects an average of the last several days, RBC reflects an average of the last several months, and whole blood is an average of both. Serum Na, K, Mg, Ca, P, Fe were analyzed on an automated clinical chemistry analyzer (Olympus AU680, Olympus America Inc.; Centerville, Pa., USA) using commercial assays. Essential minerals were measured in RBC in all cases except for sodium, lithium, and iodine; most were also measured in whole blood and/or serum depending upon which compartment is known to have the higher concentration for that mineral. Lithium was only measured in whole blood because it is more detectable there. Iodine was measured in urine (see below) because it is more detectable and reliably measured in urine than in blood. Whole blood and packed red blood cells were collected in a potassium EDTA trace metal free (royal blue top; BD Vacutainer, Franklin Lakes, NJ). Packed red blood cells were spun for 15 minutes in a centrifuge at 1500 g (g-force), the plasma and buffy coat were removed and the remaining packed red blood cells were submitted for testing. Elemental analysis was performed after digesting an aliquot of sample using a temperature controlled microwave digestion system^1 ^(Mars5; CEM Corp; Matthews, SC), following the same procedure for nitric acid microwave digestion and sample procedure as used previously for hair [34]. The digested sample was analyzed by Inductively Coupled Plasma - Mass Spectrometry (ICP-MS) (Elan DRCII; Perkin Elmer Corp; Shelton, CT). Results were verified for precision and accuracy using controls from Doctor's Data and Seronorm whole blood controls (Sero; Billingstad, Norway).

Urine iodine was analyzed by ICP-MS using a modification of the methods reported in the Analytical section of the report by the Agency for Toxic Substances and Disease Registry (ATSDR 2004). Urine results are expressed per gram creatinine.

##### Amino Acids

After an overnight fast blood samples were collected into purple top (EDTA) tubes. Blood was centrifuged within 30 minutes, and plasma was mixed with 5-sulfosalicylic acid to precipitate proteins prior to freezing for 24 hours prior to shipping. Plasma amino acids were analyzed by a reversed phase high performance liquid chromatography (HPLC) tandem mass spectrometry (MS/MS) technique (Prostar 420 HPLC autosampler, Prostar 210 solvent delivery module, 1200 L mass spectrophotometer, Varian, Inc.; Palo Alto, CA) using a method developed at Doctor's Data. Results were verified for precision and accuracy using in-house controls and a Native (Physiological) Sample Standard (Pickering Laboratories). Note that the measurement process results in oxidation of any cysteine, so that the measurement of "cysteine + cystine" is actually a measure of the combination of cysteine and cystine. The same is true of homocysteine and homocystine.

### Assessing Autistic Symptoms and Severity

Three tools were used to assess the severity and symptoms of autism, namely the Pervasive Development Disorder Behavior Inventory (PDD-BI) [35], Autism Evaluation Treatment Checklist (ATEC) [36] and Severity of Autism Scale (SAS) [37]. For the PDD-BI, a modified Autism Composite was used, following the example of a previous study [37]. That is, the Semantic/Pragmatic Problems (SemPP) subscale was omitted as children with no spoken language inappropriately score as less severe than those with limited language. The resulting modified Autism Composite consisted of Sensory/Perceptual Approach, Ritualisms/Resistance to Change, Social Pragmatic Problems, Social Approach Behaviors, Phonological and Semantic Pragmatic subscales.

### Statistical Analysis

Several types of statistical analyses were used, depending on the research question being addressed. In comparing levels between groups (such as children with autism vs. neurotypical children), 2-sided unpaired t-tests were used. The unpaired t-tests were either done assuming equal variance or unequal variance, based on the results of a test for equal variance. For individual comparisons a p value of 0.05 or lower was assumed significant. However, in order to maintain an overall significance of 5% when multiple comparisons were considered, a smaller per-test p-value was considered significant based on a Bonferroni analysis, and this p-value is specified at the beginning of each of the result sections. For example, if making 5 comparisons, then an overall significance of 5% is achieved if the p-value is set at 0.05/(5 comparisons) = 0.01. We use the term "marginally significant" if the p value is less than 0.1/(number of comparisons). We use the term "possibly significant" if the p-value is less than 0.05 but not low enough to be marginally significant; this means that the result would be significant if only one comparison were made, but could be a statistical fluke due to the making of many comparisons, so further studies are needed to confirm or invalidate the result.

Some of the data for essential minerals were not normally distributed, so in those cases a non-parametric Wilcoxon test was used instead of a t-test. Pearson correlation coefficients were obtained to determine the strengths of linear relationships among the variables involved in the analyses.

Note that for a few measurements there was some data below the detection limit. In those cases the value of the detection limit was substituted for the data point; so, for cases where some samples were below detection limit, our reported measured values are an upper bound to the true value.

Correlation and regression analysis was employed to examine the relationship between the severity of autism (assessed by the ATEC, PDD-BI, and SAS) and the biomarkers of nutritional and metabolic status. For the selected dependent and independent variables, step-wise linear regression analyses were conducted: initially all independent variables were included in the regression; then at each step, the variable with the highest p-value was eliminated, and this process was continued until the adjusted R^2 ^value began declining. Thus, the goal was to determine the best fit to the sample data for the selected model, taking into account the correlation among the independent variables. Since the data had several missing values (due to missing lab or behavioral data), the regression analyses were conducted by restricting the analysis to "complete cases" only (i.e., where there were no missing values for any of the variables in the initial analysis step). Due to the large number of biomarkers compared to the number of participants, the regression analyses were first conducted by category; for example, vitamins vs. the PDD-BI as the dependent variable. After determining, for each category, the few within-category biomarkers that had the greatest association with autism severity, an "overall" step-wise regression was performed on those biomarkers with the greatest association with autism severity. Since that "overall" analysis involved a large number of variables compared to the number of participants, the overall analysis needs to be interpreted cautiously.

## Results

### Correlation Of Autism Severity Scales

As shown in Table 4, the PDD-BI, ATEC, and SAS scales were strongly correlated with one another, R = 0.75-0.81, similar to the findings of a previous study [37].

**Table 4:** Correlations of autism severity scales

	Modified PDD-BI-Autism	ATEC	SAS
Modified PDD-BI-Autism Composite	1		

ATEC	0.81	1	

SAS	0.78	0.75	1

### Comparison of Neurotypical and Autism Groups with Published Reference Ranges

Reference ranges for the neurotypical children in this study were calculated based on the 10^th ^and 90^th ^percentiles of their distribution. This is more exact than using +/- two standard deviations if the data is not normally distributed, which sometimes was the case. These calculated reference ranges were compared with published reference ranges for vitamins (Table 5), minerals (Table 6), primary amino acids, and secondary amino acids. Two primary sources were used for vitamins and minerals: 1) the National Health and Nutrition Examination Survey (NHANES) National Report on Biochemical Indicators of Diet and Nutrition in the US Population 1999-2002 [38], and 2) the Tietz Textbook of Clinical Chemistry [39]; both are generally viewed as highly credible sources for the US population. In some cases only adult reference ranges are available from those sources. Despite the differences in techniques and methodologies, the agreement with the NHANES reference ranges is very good, and the agreement with the Tietz reference ranges is reasonable, especially when comparing to pediatric values. The agreement with published reference ranges is a validation of our methodology and of our calculated reference range for neurotypical children, which we will compare with the autism group in the next section. The advantage of having our own reference range for neurotypical children is that it closely matches the age, gender, and geographical area (Arizona) of our autism group.

**Table 5:** Vitamins: The average levels of vitamins measured in the Autism and Neurotypical groups are reported below, along with their standard deviations

Vitamins	Units	Autism Group	Neuro-typical Group	% Difference	p-value	Neurotypical Reference Range (10^th ^and 90^th ^percentiles)	Autism Group % below RR	Autism Group % above RR	**Tietz Reference Range **[39]	**NHANES1999-2002 Reference Range (6-11 yr and 12-19 yr) **[38]	Sonora Quest
Vit. A (plasma)	μg/100 ml	54.3 +/- 10.7	54.9 +/- 12		n.s.	39-71	0%	9%	26-61 (7-19 yr)	26.0-51.2 30.8-70.6 (in serum)	26-49 (7-12 yr, in serum)

Total Carotenes (beta carotene and other carotenes, in plasma)	μg/100 ml	**150** **+/- 55**	**178** **+/-53**	**-16%**	**0.01**	111-251	**29%**	8%			

Vit B1 Thiamine (WB)	μg/l	64 +/- 10	63 +/-9		n.s.	48-72	11%	20%	56 +/- 12 (children and young adults)^a^		87-280 nmol.L (adult)

Vit B2 Riboflavin (WB)	μg/l	284 +/- 42	282 +/-52		n.s.	224-332	4%	15%			

Vit B3 Niacin and Niacinamide (WB)	μg/l	7.00 +/- 1.1	7.07 +/-0.97		n.s.	5.9-8.2	16%	15%			

Vit B5 Pantothenic Acid (WB)	μg/l	**640** **+/- 128**	**714** **+/-180**	**-11%**	**0.02**	504-965	11%	0%	200-1800 (adult)		

Vit B6 (as P5P in RBC)	μg/l	17.9 +/- 16	15.2 +/-5.3		n.s.	8-21	13%	20%			

Folic Acid (serum)	μg/l	17.7 +/- 7.2	18.7 +/-6.1		n.s.	12-28	20%	7%	3-20 (adult)	9.9-33.2 6.0-24.7	3.1-17.5 (adult)

Vit B12 (plasma)	ng/l	699 +/- 235	676 +/-215		n.s.	327-938	4%	18%	200-835 (adult)	369-1260 267-941 (in serum)	243-394 (adult, in serum)

Vit C (plasma)	mg/100 ml	**1.57** **+/- 0.61**	**1.33** **+/-0.46**	**+18%**	**0.03**	0.75-1.85	13%	**29%**	0.4-1.5 (adult)	0.2-1.7 (12-19 yr, in serum)^b^	0.2-1.9 (adult)

Vit D3 (25-hydroxy in plasma)	μg/l	29.9 +/-8.4	28.6 +/-8.4		n.s.	19-44	9%	7%	14-60 (adult)	17-35 13-35	30-100

Total Vit E (serum)	mg/100 ml	**0.78** **+/- 0.18**	**0.90** **+/-0.32**	**-14%**	**0.03**	0.6-1.4	9%	0%	0.45-0.95 (1-19 yr)	0.55-1.13 0.53-1.14	

Biotin (WB)	ng/l	**394** **+/-100**	**491** **+/-164**	**-20%**	0.001 *	257-709	7%	0%	200-500 (adult)		

Vit K (plasma)	ng/l	294 +/- 158	295 +/- 189		n.s.	129-530	9%	7%	130-1190 (adult)		80-1160 (adult)

**Vitamin-like substances**											

Free Choline (RBC)	mg/l	6.3 +/- 2.9	5.6 +/- 1.7		n.s.	4.0-7.6	22%	**35%**			

Total Choline (RBC)	mg/l	**363** **+/- 66**	**310** **+/- 51**	**+17%**	< 0.0001 *	260-362	5%	**53%**			

Lipoic Acid (plasma)	μg/l	2.56 +/- 1.5	2.85 +/- 1.2		n.s.	1.2-4.5	5%	16%			

**Biomarkers of functional need for vitamins**											

FIGLU	μg/l	**1.99** **+/- 0.92**	**1.62** **+/- 0.72**	**+23 %**	**0.03**	0.65-2.6	5%	**27%**			

Methylmalonic Acid	mg/g-creatinine	9.0 +/- 7.3	7.5 +/- 5.0		n.s.	1.6-13.7	2%	13%			

N-methyl-nicotinamide	mg/g-creatinine	**5.0** **+/- 4.4**	**3.6** **+/- 2.3**	**+40%**	**0.04**	1.2-7.1	13%	17%			

Kryptopyroles	μg/dl	39.3 +/- 30.	35.8 +/- 15		n.s.	14-56	13%	11%			

The p-value for a t-test comparison of the two groups is also reported. If the p-value is below 0.05, then the % difference between the groups is reported, and the result is highlighted.

The table also lists the reference range of the neurotypical group (10^th ^and 90^th ^percentiles), and the % of the Autism group who are above or below the reference ranges. Note that if the groups were identical, 10% would be above and 10% would be below. Percentages above 25% are highlighted. Reference Ranges from Tietz Textbook of Clinical Chemistry (Burtis and Ashwood 1999), the NHANES National Report, and Sonora Quest are given where available.

a) From Wyatt et al 1991 [110].

b) From Schleicher et al 2009 [111], based on NHANES data from 1999-2002 [38].

* Statistically significant difference between the two groups with 95% confidence per Bonferroni analysis.

**Table 6:** Essential Minerals and non-essential minerals measured in whole blood, RBC, serum, and urine

Essential Minerals + other minerals	Units	Autism Group	Neuro-typical Group	% Difference	p-value	Neurotypical Reference Range (10^th ^and 90^th ^percentiles)	Autism Group % below RR	Autism Group % above RR	**Tietz Ref. Range **[39]	**NHANES1999-2002 Reference Range (6-11 yr and 12-19 yr) **[38]	Sonora Quest Ref. Range
Calcium-WB	mg/dl	5.9 +/- 0.4	5.8 +/- 0.3		n.s.	5.45-6.20	11%	11%			

Calcium-RBC	μg/g	**19.3** **+/-7**	**22.4** **+/- 6**	**-14%**	**0.02**	12.5-29.5	**31%**	7%			

Calcium-Serum (total)	mg/dl	9.6 +/- 0.5	9.6 +/- 0.2		n.s.	9.25-9.9	13%	9%	8.8-10.8 (2-12 yr)		8.8-10.8 (2-12 yr)

Chromium-RBC	ng/g	0.91 +/-0.5	0.80 +/- 0.4		n.s.	0.3-14	9%	16%			

Copper-WB	μg/dL	**95** **+/-11**	**89** **+/- 14**	**+7%**	**0.02**	70-108.5	0%	11%			

Copper-RBC	μg/g	**0.76** **+/-0.08**	**0.72** **+/- 0.09**	**+5%**	**0.03**	0.61-0.86	2%	13%			

Iodine-Urine	μg/mg-creatinine	0.24 +/- 0.2	0.26 +/- 0.3		n.s.	0.097-0.57	**25%**	4%		0.116-0.699 0.067-0.364	

Iron-RBC	μg/g	**891** **+/-94**	**833** **+/- 64**	**+7%**	0.0005 *	764-922	2%	**42%**			

Iron-Serum	μg/dl	83 +/- 34	87 +/- 35		n.s.	42-130	7%	7%	50-120 (child)	39-126 45-141	28-136 (6-14 yr)

Serum Ferritin	μg/l	39.1 +/- 22	36.9 +/- 17		n.s.	17-63	9%	16%	7-140 (1 - 15 yr)	11-74 8.0-115	

Lithium-WB	μg/L	**1.7** **+/-0.8**	**3.6** **+/- 6**	**-52%**	**0.006 W**	1-5.0	11%	0%			

Lithium-WB (without 3 highest neurotypical outliers #)	μg/L		**2.2** **+/- 1.1**	**-23%**	**0.006 W**	3.25-3.90	13%	7%			

Magnesium-WB	mg/dl	**3.53** **+/-0.31**	**3.64** **+/- 0.26**	**-3%**	**0.02 W**	42-54.5	18%	22%			

Magnesium-RBC	μg/g	48.9 +/-6	47.5 +/- 5		n.s.	1.8-2.2	4%	2%			40-64 (adult)

Magnesium-Serum	mg/dl	**1.95** **+/- 0.14**	**2.03** **+/- 0.15**	**-4%**	**0.02 W**	8-16	13%	15%	1.7-2.1 (6-12 yr)		1.7-2.4 (adult)

Manganese-WB	μg/L	12.1 +/4	11.6 +/- 3		n.s.	0.012-0.025	11%	20%	7.7-12.1 (adult)		

Manganese-RBC	μg/g	0.021 +/-0.007	0.018 +/- 0.005	+12%	0.07 W	1-1.8	6%	7%			

Molybdenum-WB	μg/L	1.4 +/-0.4	1.4 +/- 0.3		n.s.	0.15-0.30	7%	13%	0.8-3.3 (adult)		

Molybdenum-RBC	ng/g	0.93 +/- 0.3	0.98 +/- 0.2		n.s.	520-629	7%	**33%**			

Phosphorus-RBC	μg/g	**597** **+/-59**	**567** **+/- 43**	**+5%**	**0.004**	3.85-5.35	6%	6%			

Phosphorus-Serum	mg/dL	4.6 +/- 0.5	4.6 +/- 0.5		n.s.	73-83.5	9%	20%	4.5-5.5 (2-12 yr)		3.1-5.9 (4-12 yr)

Potassium -RBC	mEq/L	**79** **+/- 5.3**	**76.9** **+/- 4.1**	**+3%**	**0.007 W**	3.8-4.6	15%	9%			

Potassium-Serum	mEq/L	4.1 +/- 0.3	4.2 +/- 0.3		n.s.	186-236	19%	9%	3.4-4.7 (child)		2.8-6.0 (child)

Selenium-WB	μg/L	207 +/-28	210 +/- 20		n.s.	0.20-0.27	9%	18%	58-234 (adult)		

Selenium-RBC	μg/g	0.24 +/- 0.04	0.23 +/- 0.03		n.s.	136-139	9%	17%	0.07-0.24 (adult)		

Sodium-Serum	mEq/l	138 +/- 2	137 +/- 1		n.s.	0.15-0.30	11%	2%	138-145 (child)		135-145 (adult)

Vanadium-RBC	ng/g	0.21 +/- 0.07	0.22 +/- 0.07		n.s.	465-657	7%	7%			

Zinc-WB	μg/dL	551 +/-68	555 +/- 74		n.s.	6.8-10.8	2%	15%			

Zinc-RBC	μg/g	9.2 +/-1.4	8.9 +/- 1.4		n.s.						

**Non-essential minerals**											

Boron-RBC	μg/g	**0.029** **+/-0.014**	**0.025** **+/- 0.007**	**+16%**	**0.04**	0.014-0.032	13%	**36%**			

Strontium-WB	μg/L	25 +/-8	24 +/- 6		n.s	17-34	7%	6%			

The average levels of minerals measured in the Autism and Neurotypical groups are reported below, along with their standard deviations. The p-value for a t-test comparison of the two groups is also reported. If the p-value is below 0.05, then the % difference between the groups is reported. For several tests the data was not normally distributed, and in those cases a non-parametric Wilcoxon test (also known as the Mann-Whitney test) was used instead of a t-test - those p-values are marked with a W.

The table also lists the reference range of the neurotypical group (10^th ^and 90^th ^percentiles), and the % of the Autism group who are above or below the reference ranges. Note that if the groups were identical, 10% would be above and 10% would be below. Percentages above 25% are highlighted. Reference Ranges from Tietz Textbook of Clinical Chemistry (Burtis and Ashwood 1999), the NHANES National Report, and Sonora Quest are given where available.

# Note that the lithium level for the neurotypical group is greatly increased by three siblings with very high lithium levels of 16, 16, and 35 mcg/L. A second value for lithium is given for the neurotypical group without those 3 siblings; the difference between the autism group and this revised neurotypical group is smaller but slightly more significant.

* Statistically significant difference between the two groups with 95% confidence per Bonferroni analysis.

The amino acid reference ranges (10^th ^and 90^th ^percentiles) for the neurotypical group (present study) were compared with pediatric reference ranges by Lepage et al [33] and (where available) with values from the Tietz Textbook of Clinical Chemistry [39]. For Lepage et al [33], two reference ranges are listed, one for 6 year olds and one for 16 year olds. In most cases the values from the present study are in reasonable agreement with the published values.

For the autism group, the average (mean) levels of their vitamins, minerals, and most amino acids were within the published reference ranges (where available). However, as will be discussed below, a t-test comparison of the levels of vitamins, minerals, amino acids, and other biomarkers in the autism group and the neurotypical group revealed many significant differences.

### Comparisons of biomarkers between autistic and neurotypical groups

#### Vitamins

**Figure 1:**
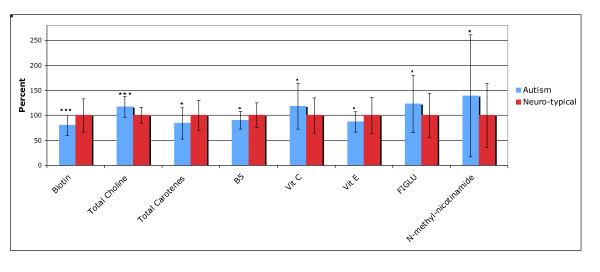
**Vitamins and related substances which were significantly different between the autism and neurotypical groups, rescaled to the average neurotypical values**. The average values and the standard deviations are shown. The number of asterisks indicates the p-value (* p < 0.05, ** p < 0.01, *** p < 0.001).

Table 5 shows the participants' levels of vitamins, vitamin-like substances, and biomarkers of vitamin status. Because we are making multiple comparisons (our hypothesis is "are the levels of any vitamins different in children with autism vs. controls"), we need to apply a Bonferroni correction (see statistical analysis section). For 21 comparisons, p values are defined as: "significant" = p < 0.002, "marginally significant" = p < 0.005, and "possibly significant" = p < 0.05. Figure 1 compares the levels of vitamin-related biomarkers that were different in the autism group compared to the control group.

For the vitamins, the only significant difference was a 20% lower biotin (p < 0.001) in the children with autism. There were possibly significant (p < 0.05) lower levels of vitamin B5, vitamin E, and total carotenoids. Vitamin C was possibly slightly higher in the children with autism. Vitamin B6 (measured as the active form, P5P, in the RBC) had an unusually broad distribution in children with autism compared to controls (see Figure 1), with the levels in the children with autism having 3 times the standard deviation of the neurotypical children.

The levels of two vitamin-like substances, lipoic acid and choline (free and total) were also assessed. Levels of lipoic acid and free choline were similar in the two groups, but total choline was 17% higher in the autistic group (p < 0.0001).

The functional need for vitamins was indirectly assessed by measurements of several urinary metabolites, including FIGLU, kryptopyroles, methylmalonic acid, and n-methyl-nicotinamide. FIGLU and n-methyl-nicotinamide were somewhat higher in children with autism (possibly significant, p < 0.05), suggesting an increased need for folic acid and niacin, respectively. The average levels of urinary kryptopyroles were not significantly different in children with autism, but the children with autism had a much broader distribution.

For most vitamins, children with autism have levels that lie within the neurotypical reference ranges defined by the 10^th ^and 90^th ^percentiles (see Table 5). However, there are some cases where more than 25% of the autism group lie below the neurotypical reference range (total carotenes) or above the neurotypical reference range (vitamin C, free choline, total choline, FIGLU).

#### Essential Minerals

**Figure 2:**
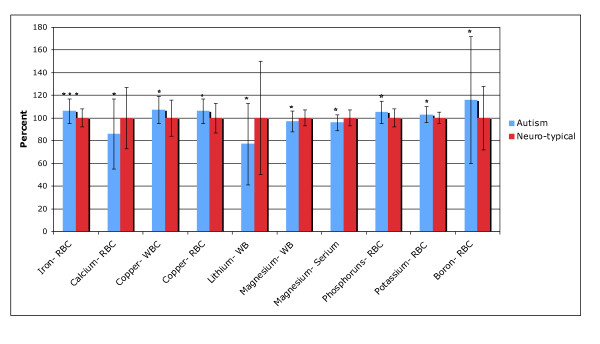
**Minerals which were significantly different between the autism and neurotypical groups, rescaled to the average neurotypical values**. The average values and the standard deviations are shown. The number of asterisks indicates the p-value (* p < 0.05, ** p < 0.01, *** p < 0.001).

Table 6 shows the levels of minerals in whole blood (WB), red blood cell (RBC), serum, and urine (for iodine) for the study participants. (28 comparisons: "significant" is p < 0.002, "marginally significant" is p < 0.004, and "possibly significant" is p < 0.05). Figure 2 shows the levels of minerals which were different between the autism and neurotypical groups.

The largest difference was a much lower level of WB lithium (-53%, p < 0.006). Note that three of the controls had unusually high levels of WB lithium, and were from the same family, so the data is analyzed with and without their data; removing their results reduces the magnitude of the difference, but the significance of the result remains the same.

Iron status was measured in three ways: serum ferritin, serum iron, and RBC iron. The first two did not reveal any difference between the two groups, but RBC iron was slightly higher in the children with autism (+7%, p < 0.0005), with 42% of the children with autism having levels above the 90^th ^percentile for the typical children.

There were small, possibly significant differences in several other minerals. There were possibly significant slightly higher levels of RBC potassium, RBC phosphorus, copper (WB and RBC), and RBC boron, and a possibly significant lower level of RBC calcium and magnesium (serum and WB).

For most minerals, children with autism have levels that generally lie within the neurotypical reference ranges (see Table 6). However, there are some cases where more than 25% of the autism group lie below the neurotypical reference range (urinary iodine, RBC calcium) or above the neurotypical reference range (RBC iron, RBC phosphorus, RBC boron).

We also investigated the correlations of levels of minerals measured in different blood compartments, as shown in Table 7. In some cases the levels correlate strongly, but in some they do not; in the latter case measurements for those elements need to be interpreted cautiously as different compartments will give different results. For magnesium, copper, zinc, manganese, and selenium there are strong, very significant correlations of levels between the WB and RBC, and a modest correlation for molybdenum. For calcium and magnesium, there is a significant correlation of levels in WB and serum. For calcium, there is a small negative correlation of RBC and serum levels. For potassium and phosphorus, correlations between RBC and serum are generally not significant, except possibly for a weak correlation for phosphorus for the neurotypical group. For iron, there are no significant correlations between levels in RBC iron, serum iron, and serum ferritin. In summary, interpretation of results for some elements (magnesium, copper, zinc, manganese, and selenium) is consistent across blood compartments, but for some elements it is not (calcium, potassium, phosphorus, iron).

**Table 7:** Correlations of essential minerals in different blood components

	Comparison	Autism Group	Neurotypical Group
**Calcium**	WB -RBC	0.00	0.14

	WB-Serum	0.40	0.33

	RBC-Serum	-0.30	-0.32

**Magnesium**	WB-RBC	0.52	0.65

	WB-Serum	0.41	0.34

	RBC-serum	0.37	0.13

**Copper**	WB-RBC	0.46	0.74

**Zinc**	WB-RBC	0.65	0.84

**Manganese**	WB-RBC	0.84	0.90

**Selenium**	WB-RBC	0.79	0.62

**Molybdenum**	WB-RBC	0.45	0.24

**Potassium**	RBC-Serum	-0.18	0.14

**Phosphorus**	RBC-Serum	0.11	0.28

**Iron**	RBC-Serum	-0.03	0.13

	RBC-serum ferritin	-0.09	0.13

	Serum- serum ferritin	-0.08	-0.24

#### Sulfation, Methylation, Glutathione, Oxidative Stress

**Figure 3:**
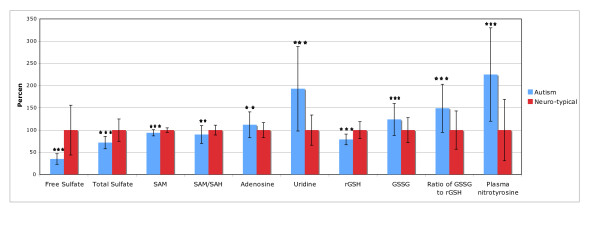
**Sulfation, methylation, glutathione, and oxidative stress biomarkers which were significantly different between the autism and neurotypical groups, rescaled to the average neurotypical values**. The average values and the standard deviations are shown. The number of asterisks indicates the p-value (* p < 0.05, ** p < 0.01, *** p < 0.001).

Table 8 shows the results for sulfation, methylation, glutathione, and oxidative stress markers. (11 comparisons, so "significant" is p < 0.005, "marginally significant" is p < 0.01, and "possibly significant" is p < 0.05). Figure 3 shows the results which were different between the autism and neurotypical groups.

**Table 8:** Metabolic Markers The average levels measured in the Autism and Neurotypical groups are reported below, along with their standard deviations

	Units	Autism Group	Neuro-typical Group	% Difference	p-value	Neurotypical Reference Range (10^th ^and 90^th ^percentiles)	Autism Group % below RR	Autism Group % above RR
Free Sulfate (plasma)	μmol / g protein	**1.44** **+/- 0.51**	**4.09** **+/- 2.28**	**-65%**	< 0.00001 *	1.4-7.5	**56%**	0%

Total Sulfate (plasma)	μmol / g-protein	**1121** **+/- 212**	**1566** **+/- 384**	**-28%**	< 0.0001 *	987-2070	**36%**	0%

SAM (RBC)	μmol/dl	**214.5** **+/- 15**	**228.4** **+/- 12**	**-6%**	< 0.0001 *	210-242	**39%**	4%

SAH (RBC)	μmol/dl	44.6 +/- 8.0	42.6 +/- 4.4		n.s.	40-52	**27%**	24%

SAM/SAH ratio		**4.9** **+/- 1.1**	**5.4** **+/- 0.6**	**-10%**	**0.006**	4.1-6.0	**25%**	13%

Uridine (plasma)	10^-6 ^mol/l	**15.3** **+/- 7.5**	**7.9** **+/- 2.7**	**+93%**	< 0.00001 *	5.5-10.9	4%	**60%**

Adenosine (plasma)	10^-8 ^mol/l	**23.2** **+/- 5.9**	**20.6** **+/- 3.4**	**+12%**	**0.008**	17-26	11%	**33%**

Inosine (plasma)	10^-6 ^mol/l	3.56 +/- 0.91	3.83 +/- 0.93		n.s.	2.7-5.2	16%	5%

Reduced plasma glutathione (GSH)	nmol/ml	**3.23** **+/- 0.48**	**4.09** **+/- 0.79**	**-21%**	< 0.0001 *	3.1-5.1	**53%**	0%

Oxidized glutathione (GSSG)	nmol/ml	**0.447** **+/- 0.13**	**0.362** **+/- 0.10**	**+24%**	0.001 *	0.22-0.52	0%	**30%**

Ratio of oxidized to reduced plasma glutathione		**0.14** **+/- 0.05**	**0.093** **+/- 0.04**	**+49%**	< 0.0001 *	0.05-0.15	4%	**42%**

Plasma nitro-tyrosine	μg/l	**16.6** **+/- 7.8**	**7.4** **+/- 5.1**	**+125%**	< 0.0001 *	3.7-18	0%	**44%**

The p-value for a t-test comparison of the two groups is also reported. If the p-value is below 0.05, then the % difference between the groups is reported, and the result is highlighted.

The table also lists the reference range of the neurotypical group (10^th ^and 90^th ^percentiles), and the % of the Autism group who are above or below the reference ranges. Note that if the groups were identical, 10% would be above and 10% would be below. Percentages above 25% are highlighted.

* Statistically significant difference between the two groups with 95% confidence per Bonferroni analysis.

Free and total sulfate in plasma were very significantly lower in children with autism (-28% and -65%, respectively, p < 0.0001).

S-adenosylmethionine (SAM, the primary methyl donor in the body) was also very significantly lower in children with autism vs. controls. Although the percentage difference is not high, the normal reference range is very narrow, so this difference is very significant. The level of SAH was not significantly different, but it had an unusually broad distribution, with 27% of the children with autism having levels below the 10^th ^percentile of the neurotypical group, and 24% had levels above the 90^th ^percentile. The SAM/SAH ratio was 10% lower in children with autism (p = 0.006).

Uridine (in plasma) was very significantly higher in the children with autism (+93%, p < 0.0001). Uridine is believed to be a marker of methylation status, and in fact SAM and uridine were somewhat negatively correlated (R = -0.30).

Adenosine was slightly higher (marginally significant) in children with autism, which may indicate that some children have an impairment in adenosine deaminase.

Reduced plasma glutathione (GSH) was very significantly lower in the children with ASD. GSH is an important anti-oxidant and important for excretion of toxic metals.

All three markers of oxidative stress, namely oxidized glutathione (GSSG), the ratio of oxidized to reduced glutathione (GSSG:GSH), and plasma nitrotyrosine, were very significantly higher in children with autism.

For sulfation, 36-56% of the autism group have sulfate levels below the neurotypical reference range (see Table 8). For SAM, SAH, and SAM/SAH, 25-39% of the autism group have low levels, and 60% have elevated uridine, another marker of methylation status. Adenosine was elevated in 33% of the autism group. For reduced glutathione, oxidized glutathione, the ratio of GSH:GSSG, and nitrotyrosine, 30-53% of the autism group have abnormal values.

#### ATP, NADH, NADPH, CoQ10

**Figure 4:**
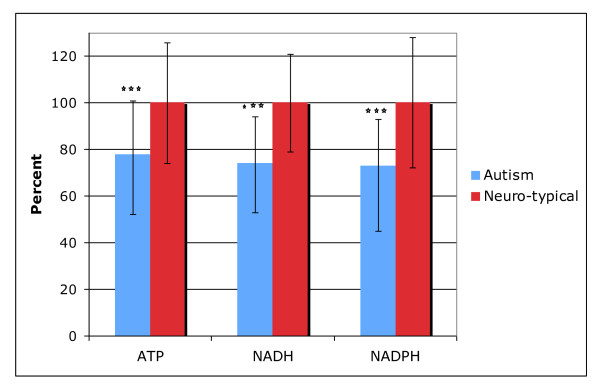
**ATP, NADH, and NAHPH were significantly different between the autism and neurotypical groups**. The average values and the standard deviations are shown, rescaled to the average neurotypical value. The number of asterisks indicates the p-value (* p < 0.05, ** p < 0.01, *** p < 0.001).

Table 9 shows the results for ATP, NADP, NADPH, and CoQ10. (4 comparisons: "significant" is p < 0.012, "marginally significant" is p < 0.025, and "possibly significant" is p < 0.05). Figure 4 shows the results which were different between the autism and neurotypical groups.

**Table 9:** ATP, NADH, NADPH, and CoQ10 The average levels measured in the Autism and Neurotypical groups are reported below, along with their standard deviations

	Units	Autism Group	Neuro-typical Group	% Difference	p-value	Neurotypical Reference Range (10^th ^and 90^th ^percentiles)	Autism Group % below RR	Autism Group % above RR
ATP (plasma)	nmol/l	**14.5** **+/- 4.2**	**18.5** **+/- 4.8**	**-22%**	< 0.0001 *	13.21	**36%**	4%

NADH (RBC)	nmol/ml	**15.3** **+/- 4.1**	**20.7** **+/- 4.3**	**-26%**	< 0.0001 *	16-25	**51%**	2%

NADPH (RBC)	nmol/ml	**22.6** **+/- 6.1**	**30.9** **+/- 8.5**	**-27%**	< 0.0001 *	20-40	**37%**	2%

CoQ10 (plasma)	μg/ml	0.55 +/- 0.15	0.60 +/- 0.16		n.s.	0.4-0.8	9%	2%

The p-value for a t-test comparison of the two groups is also reported. If the p-value is below 0.05, then the % difference between the groups is reported, and the result is highlighted.

The table also lists the reference range of the neurotypical group (10^th ^and 90^th ^percentiles), and the % of the Autism group who are above or below the reference ranges. Note that if the groups were identical, 10% would be above and 10% would be below. Percentages above 25% are highlighted.

* Statistically significant difference between the two groups with 95% confidence per Bonferroni analysis.

The primary function of mitochondria is to produce ATP, the primary energy source in the brain and in the body. CoQ10 is an important co-factor for mitochondrial function. We found that children with ASD have levels of plasma CoQ10 that are very similar to the neurotypical group. Levels of CoQ10 did not significantly correlate with levels of ATP or with autism severity. The autism group had much lower levels of plasma ATP and of NADH (RBC) and NADPH (RBC), which are the precursors to ATP, and 36-51% of the autism group had levels below the neurotypical reference range. The level of ATP, NADH, and NADPH were all highly correlated with one another (r = 0.67-0.69, p < 0.001).

#### Plasma Amino Acids: Primary

**Figure 5:**
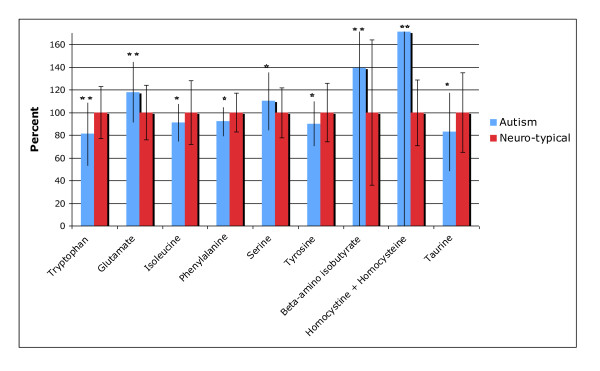
**Amino Acids which were significantly different between the autism and neurotypical groups, rescaled to the average neurotypical value**. The average values and the standard deviations are shown. The number of asterisks indicates the p-value (* p < 0.05, ** p < 0.01, *** p < 0.001). The standard deviations for beta-amino-isobutyrate and "homocystine + homocysteine" are outside the margins of the figure.

The levels of primary (proteinogenic) plasma amino acids are given in Table 10. Note that these are free, not total, amino acids in plasma. (20 comparisons: "significant" is p < 0.0025, "marginally significant" is p < 0.005, and "possibly significant" is p < 0.05). Figure 5 shows the results which were different between the autism and neurotypical groups.

**Table 10:** Table of Primary Amino Acids in Plasma The average levels measured in the Autism and Neurotypical groups are reported below, in units of μmol/dl, along with their standard deviations

Amino Acids	Autism Group	Neuro-typical Group	% Difference	p-value	Neurotypical Reference Range (10^th ^and 90^th ^percentiles)	Autism Group % below RR	Autism Group % above RR	Tietz Ref. Range	Lepage et al Reference Range at 6 yr (top) and 16 yr (bottom)
**Essential Amino Acids**									

Histidine	8.84 +/- 2.0	8.2 +/- 1.3		0.06	6.4-9.8	15%	**36%**		6.3-9.3 7.7-10.7

Isoleucine	**5.26** **+/- 1.0**	**5.8** **+/- 1.6**	**-9%**	**0.05**	4.1-8.3	16%	0%	3.8-9.5 (6-18 yr, serum)	4.0-6.9 4.7-7.4

Leucine	10.7 +/- 2.2	10.7 +/- 2.1		n.s.	8.2-13	13%	11%	7.9-17.4 (6-18 yr, serum)	8.6-13.6 10.1-15.9

Lysine	13.5 +/- 3.8	14.5 +/- 4.8		n.s.	8.7-22	11%	2%		9.6-18.1 15.7-24.2

Methionine	1.83 +/- 0.46	1.75 +/- 0.34		n.s.	1.4-2.3	16%	13%	1.6-3.7 (6-18 yr, serum)	1.4-2.5 2.0-3.4

Phenylalanine	**4.45** **+/- 0.62**	**4.83** **+/- 0.83**	**-8%**	**0.01**	3.9-6.0	20%	0%	4.8-10.9 (adult, serum)	4.0-6.1 4.7-7.4

Threonine	9.25 +/- 2.9	8.88 +/- 2.1		n.s.	6.7-11	18%	16%		6.5-12.5 10.4-18.8

Tryptophan	**3.49** **+/- 1.2**	**4.33** **+/- 1.0**	**-19%**	0.001 *	2.7-5.6	24%	4%		3.7-7.6 5.4-9.3

Valine	19.5 +/- 3.8	20.5 +/- 4.3		n.s.	16-27	16%	4%	15.6-28.8 (6-18 yr, serum)	16.5-23.4 17.8-27.5

**Other Amino Acids**									

Alanine	36.4 +/-10.5	33.4 +/- 8.9		n.s.	24-45	7%	18%	19.3-54.5 (6-18 yr, serum)	18.2-31.9 24.0-48.2

Arginine	6.50 +/- 2.4	6.7 +/- 1.8		n.s	4-9.35	16%	9%		5.0-9.9 6.8-12.8

Asparagine	4.33 +/- 1.0	4.40 +/- 0.85		n.s.	3.6-5.7	**30%**	8%		3.1-6.7 3.7-8.1

Aspartate	0.71 +/-0.29	0.78 +/- 0.38		0.08	0.39-1.25	8%	6%		0.3-0.6 0.2-0.5

Cystine (oxidized form of cysteine)	3.22 +/- 0.82	3.48 +/- 0.74		n.s.	2.4-4.25	15%	11%		

Glutamate	**6.5** **+/- 1.5**	**5.5** **+/- 1.3**	**+18%**	0.001 *	4.2-7.7	6%	**26%**		1.3-6.5 1.1-4.6

Glutamine	43.3 +/- 9.0	41.6 +/- 6.9		n.s.	34-52	16%	20%	36-74 (6-18 yr, serum)	49.3-72.4 55.1-79.7

Glycine	26.7 +/- 8.1	27.3 +/- 10.1		n.s.	16-39	4%	6%		14.4-28.2 18.3-32.2

Proline	15.7 +/- 5.5	15.8 +/- 4.9		n.s.	11.5-24	20%	7%		9.3-20.1 11.3-27.1

Serine	**10.4** **+/- 2.5**	**9.47** **+/- 2.1**	**+10%**	**0.04**	6.9-12	5%	15%		9.6-15.5 10.1-17.7

Tyrosine	**5.51** **+/- 1.2**	**6.1** **+/- 1.6**	**-10%**	**0.03**	4.8-8	**31%**	4%	4.4-7.2 (adult)	3.9-6.5 4.6-8.7

The p-value for a t-test comparison of the two groups is also reported. If the p-value is below 0.05, then the % difference between the groups is reported, and the result is highlighted.

The table also lists the reference range of the neurotypical group (10^th ^and 90^th ^percentiles), and the % of the Autism group who are above or below the reference ranges. Note that if the groups were identical, 10% would be above and 10% would be below. Percentages above 25% are highlighted. Reference Ranges from Tietz Textbook of Clinical Chemistry are given for serum amino acids where available. For Lepage et al, the reference ranges are the 10^th ^and 90^th ^percentiles for ages 6 yr and ages 16 yr.

* Statistically significant difference between the two groups with 95% confidence per Bonferroni analysis.

A few samples had abnormally low ratios of glutamine/glutamate (< 4) and also asparagine/aspartate (< 5). These amino acids are especially sensitive to shipping conditions, and abnormalities in both ratios suggest that some thermal degradation occurred during shipping/processing, resulting in conversion of some glutamine to glutamate, and asparagine to aspartate. This was the case for five autism samples and three control samples. The results for those samples were not included in the analysis.

The autism group had significantly lower levels of tryptophan, a precursor to serotonin, (-19%, p < 0.001) and higher levels of glutamate, an excitatory neurotransmitter (+18%, p < 0.001). There were smaller changes in other amino acids that were possibly significant (p < 0.05), including slightly increased serine, and slightly decreased tyrosine and phenylalanine.

For most primary amino acids, children with autism had levels that generally lay within the neurotypical reference ranges (see Table 10). However, there are some cases where more than 25% of the autism group lie below the neurotypical reference range (asparagine, tyrosine) or above the neurotypical reference range (histidine, glutamate).

#### Plasma Amino Acids: Secondary

The levels of secondary plasma amino acids and amino acid metabolites are given in Table 11. Cystathionine was also measured, but all the measurements except 1 were below the detection limit of 0.05 umoles/100 ml, so those values are not listed. (21 comparisons: "significant" is p < 0.002, "marginally significant" is p < 0.005, and "possibly significant" is p < 0.05). Figure 5 shows the results which were different between the autism and neurotypical groups.

**Table 11:** Secondary Plasma Amino Acids and their metabolites, in units of μmol/dl

Amino Acids	Autism Group	Neuro-typical Group	% Difference	p-value	Neuro-typical Ref. Range (10^th ^and 90^th ^percentiles)	Autism Group % below RR	Autism Group % above RR
1-Methyl histidine	0.365 +/- 0.15	0.355 +/- 0.12		n.s.	0.19-0.52	11%	15%

3-Methyl histidine	0.74 +/- 0.68	0.68 +/- 0.52		n.s.	0.20-1.4	13%	20%

Alpha-amino adipate (33%/27% below dl)	0.079 +/- 0.32	0.088 +/- 0.044		n.s.	0.05-0.17	0%	0%

Alpha-amino-N-butyrate	1.78 +/- 0.66	1.82 +/- 0.72		n.s.	1.0-2.8	11%	7%

Anserine (85%/95% below dl)	0.058 +/- 0.027	0.051 +/- 0.006		0.06	0.32-1.0	11%	11%

Beta-alanine	0.60 +/- 0.28	0.62 +/- 0.31		n.s.	0.05-0.26	0%	15%

Beta-amino isobutyrate	**0.191** **+/- 0.89**	**0.138** **+/- 0.088**	**+39%**	**0.004**	2.45-3.75	**25%**	20%

Carnosine (76%/89% below dl)	0.061 +/- 0.031	0.054 +/- 0.015		n.s.	2.4-4.3	15%	11%

Citrulline	3.02 +/- 0.80	3.02 +/- 0.52		n.s.	0.31-1.55	9%	18%

Cystine + Cysteine	3.22 +/- 0.82	3.48 +/- 0.74		n.s.	1.3-3.6	5%	5%

Ethanolamine	1.07 +/- 0.75	0.94 +/- 0.63		n.s.	0.13-0.65	2%	9%

Gamma-amino butyrate (75%/84% below dl)	0.053 +/- 0.006	0.053 +/- 0.008		n.s.	3.9-8.3	9%	5%

Homocystine + Homocysteine (71%/89% below dl)	**0.0094** **+/- 0.010**	**0.0055** **+/- 0.0016**	**+71%**	**0.006**	0.87-2.2	**25%**	13%

Hydroxy proline	2.40 +/- 0.80	2.19 +/- 0.80		n.s.	0.0085-0.022	5%	**33%**

Methionine Sulfoxide	0.368 +/- 0.20	0.315 +/- 0.21		n.s.	0.4-1.35	13%	5%

Ornithine	5.9 +/- 1.6	5.98 +/- 1.7		n.s.	5.3-23.5	4%	4%

Phospho ethanol amine	1.34 +/- 0.73	1.55 +/- 0.59		n.s.	195-400	2%	3%

Phospho serine	0.045 +/- 0.078	0.024 +/- 0.044		n.s.			

Sarcosine	0.80 +/- 0.32	0.893 +/- 0.36		n.s.			

Taurine	**13.9** **+/- 5.8**	**16.8** **+/- 5.8**	**-17%**	**0.02**			

Urea	299 +/- 94	309 +/- 87		n.s.			

In some cases data was below the detectable limit -if this was greater than 20%, then we report the data as the % of the autism group and then the % of the neurotypical group below the detectable limit (dl).

The table also lists the reference range of the neurotypical group (10^th ^and 90^th ^percentiles), and the % of the Autism group who are above or below the reference ranges. Note that if the groups were identical, 10% would be above and 10% would be below. Percentages above 25% are highlighted.

The autism group had a higher level of beta-amino isobutyrate (+39%, p = 0.004, marginally significant). They had a possibly significant lower level of taurine. They also had a possibly significant much higher level of "homocystine + homocysteine"- however, it should be noted that 71% of the autism group and 89% of the neurotypical group had levels below the detectable limit, so the "homocystine + homocysteine" results should be interpreted with caution.

For the secondary amino acids, children with autism had levels that generally lay within the neurotypical reference ranges (see Table 11). However, there were some cases where more than 25% of the autism group lay below the neurotypical reference range (citrulline, phosphoethanolamine) or above the neurotypical reference range (phosphoserine).

### Medication Effects

45% of the children with autism were taking one or more medications (see Table 1). Some of those medications may have affected their levels of vitamins, minerals, or other biomarkers. Some psychopharmaceuticals (such as risperidone and clonidine used by several of this ASD population) and anti-convulsants (valproic acid and topiramate used by two of the ASD subjects) are known to interfere with nutrient levels and gastrointestinal function in a variety of ways. Anticonvulsants may interfere in energy production [40] and folate metabolism and for some in levels of vitamin D. Valproate, a known teratogen [41,42] and hepatotoxin [42] as studied in humans and animals increases GABA in the brain and is a folate antagonist with absorption not affected. In animal studies [43] valproate has been shown to increase oxidative stress as methionine and vitamin E mitigate teratogenic effects. Valproate inhibits histone deacetylases, increasing accessibility of DNA to demethylases resulting in altered gene expression. In humans valproate may increase plasma ammonia, homocysteine and glutamine and decrease carnitine [42]. Topiramate may create metabolic acidosis, decrease glutamate and increase GABA. The effect upon specific nutrients has not been as well studied for the psycho-pharmaceuticals [44].

To investigate if medication use had a significant effect on results, a t-test comparison was made between the autism group taking medications (45%) vs. the autism group not taking any medications (55%). The only differences with a p-value less then 0.01 were lower RBC copper (-9% lower, p = 0.001) and higher plasma methionine sulfoxide (+35% higher, p = 0.002) for the autism medication group compared to the autism no-medication group. So, aside from those two differences, it appears that medication use had little effect on the results.

### Correlations with Autism Severity

The correlations of each biomarker with each of the three autism severity scales were calculated. Table 12 lists the biomarkers which had the highest correlation with autism severity (r > 0.34 in absolute magnitude, corresponding to a p value of 0.01 or lower). The biomarkers had p < 0.01 for only one autism severity scale at most. Given multiple biomarkers, the cut-off for significance is below p = 0.001. So, none of the results are significant, but some have p < 0.01 and are worth further investigation.

**Table 12:** Correlation of Biomarkers with the modified PDD-BI Autism Composite, ATEC, and SAS, with the correlation coefficient followed by the p-value in parentheses

	PDDBI	ATEC	SAS
Plasma Free Sulfate	-0.19 (n.s.)	-0.25 (n.s.)	**-0.38 (0.006)**

RBC Iron	**0.36 (0.009)**	0.27 (0.05)	0.12 (n.s.)

Serum Phosphorus	**0.38 (0.006)**	0.11(n.s.)	0.12 (n.s.)

Plasma Phenylalanine	-0.22 (n.s.)	-0.29 (0.03)	**-0.43 (0.002)**

Only biomarkers with values of R = 0.34 or greater are listed, corresponding to a p-value of 0.01 or lower. Note that since many correlations were investigated, these results are not highly significant, and are at most possibly significant.

### Regression Analyses

Regression analysis develops an equation that relates one or more "independent" variables (such as metabolic biomarkers) to a single "dependent" variable (such as severity of autism). The regression equation has coefficients that minimize the differences between observed values of the dependent variable and those predicted by the equation. The standard measure of how well a regression performs is R^2^, which is the proportion of the variation in the dependent variable that can be explained by the regression. (If R^2 ^= 1, the regression equation fits the dependent variable perfectly; if R^2 ^= 0 the independent variables provide no useful information about the dependent variable).

#### Vitamins

The regression analysis yielded a significant result for all three autism severity scales (adj. R^2 ^of 0.25-0.57), with the highest adjusted R^2 ^for the PDD-BI. Vitamin B6, Vitamin C, N-methyl-nicotinamide, and Vitamin K were the most consistently significant variables.

#### Minerals

The regression analysis yielded a significant result for all three scales (adj. R^2 ^of 0.22-0.38), with the highest adjusted R^2 ^for the PDD-BI. Calcium (RBC), Iron (RBC), Zinc (WB and RBC), and Potassium (RBC) were the most consistently significant variables. Note that almost all of the most consistently significant variables were in RBC; ie, it is the RBC levels that seem to be most strongly associated with autism severity.

#### Sulfation, Methylation, Glutathione, Oxidative Stress

The regression analysis yielded significant results for all three scales, with all three severity scales having modest adjusted R^2 ^(0.15-0.24). Free Sulfate was the most consistently significant variable, followed by Oxidized Glutathione and SAM.

#### ATP, NADH, NADPH, CoQ10

The regression analysis yielded significant results for only one severity scale (the ATEC), with only a modest adjusted R^2 ^(0.15). NADH and ATP were the two significant variables.

#### Primary Amino Acids

The regression analysis yielded significant results for all three scales (adj. R^2 ^of 0.22-0.39), with the PDD-BI having the highest adjusted R^2^. Proline and Serine were the most consistently significant variables.

#### Secondary Amino Acids

The regression analysis yielded significant results for all three scales, with modest adjusted R^2 ^(0.18-0.26). Ethanolamine and Beta-amino-isobutyrate were the most consistently significant variables.

#### Overall Analysis

This analysis involved starting with all variables from the previous analyses that were significant in one or more of the subgroup analyses (p < 0.01), determined individually for each autism severity scale. The Overall regression analysis yielded highly significant results for all three scales (p < 0.002 or better for all cases), with the highest adjusted R^2 ^for the PDD-BI, followed by the SAS and then the ATEC. Different markers were significant for different autism severity scales.

## Discussion

### Overview

The general agreement of the present neurotypical reference ranges of many vitamins, minerals, and primary amino acids with published reference ranges from standard sources provides validation of the methodology used. Children with autism have mean levels of vitamins, minerals, and most primary amino acids that generally lie within published reference ranges. In the care of children with autism, the practitioner is therefore unable to discern emerging metabolic dysfunction or utilize measured values of these standard analytes as the basis for clinical decision-making regarding supplementation in most cases. More reliable and of greater clinical significance are levels of sulfation, SAM, uridine, glutathione, oxidative stress, and ATP/NADH/NADPH which are very likely to be abnormal.

In the sections below we provide a detailed discussion of each of the categories of measurements. However, some of the results are inter-related, so we wish to first discuss some of the major results.

#### Sulfation and ATP

Children with autism had significantly lower levels of plasma sulfate, including both free and total sulfate, consistent with several previous studies. ATP is required in the kidney to resorb sulphate (recycling of sulphate is important because sulphate is poorly absorbed from the gut, and conversion from cysteine is slow). This study found a significant correlation of ATP with free and total plasma sulphate (r = 0.32 and 0.44, respectively), suggesting that decreased ATP is a significant contributor to decreased sulphate levels in children with autism.

#### Methylation and ATP

Children with autism had significantly impaired methylation, as evidenced by low levels of SAM (the primary methyl donor) and high levels of plasma uridine (which requires methylation to be converted to thymidine). SAM is formed from methionine by methionine adenyosyl transferase, which requires ATP. Methionine levels were similar in the autism and neurotypical groups, but ATP levels were very significantly lower in the autism group, suggesting that low levels of ATP are at least part of the reason for decreased levels of SAM. Methionine in the body comes partially from the diet (it is an essential amino acid) and partially by the recycling of homocysteine to methionine (via methionine synthase or methyl transferase). Methionine synthase requires methyl-B12 and 5-methyl-tetrahydrofolate, a derivative of folic acid. Both vitamin B12 and folic acid were similar in the autism and control groups, consistent with normal levels of methionine. Normal levels of methionine are consistent with one previous study [10] which involved the use of age-matched controls with no intake of vitamins/minerals; previous studies of methionine levels had reported significant differences, but those studies involved either children with autism taking a vitamin/mineral supplement [8] or significant age differences [9].

#### Oxidative Stress

Children with autism had significantly elevated oxidative stress, as indicated by increased GSSG/GSH ratio (glutathione is the primary anti-oxidant in the body), and increased plasma nitrotyrosine. GSSG is reduced to GSH by glutathione reductase, which requires NADPH. NADPH levels were substantially lower in children with autism, which would explain why they also had a decreased GSH/GSSG ratio. These results are consistent with several previous studies discussed in the Introduction.

### Vitamins

The data show that, on average, children with autism have lower levels of biotin, and trend toward lower levels of vitamin B5, vitamin E, and total carotenoids. Those nutrients were also more likely to be below the reference range of the neurotypical group. The autism group had somewhat higher levels of vitamin C.

The broad distribution of vitamin B6 (measured as the active form, P5P, inside the RBC) is very interesting. Two previous studies [45,46] found high levels of total B6 in the plasma, and in those papers it was hypothesized that it might be due to an impaired conversion of B6 to the active form, P5P. That hypothesis may be valid for the subset of children with low RBC P5P, but there also appears to be a subset with high RBC P5P. Overall, the broad distribution of RBC P5P suggests that there is a subset of children who need more vitamin B6, and a subset who have high levels of B6. This is consistent with 11 double-blind, placebo-controlled studies which mostly found that about half of children or adults with autism benefit from mega-doses of vitamin B6 (500-1000 mg, or 250-500× the RDA) [45]. This study suggests that there may be a subgroup (those with low RBC P5P) who would likely benefit from B6 supplementation. However, since B6 levels are not dramatically low, it is possible that high doses (20-40× RDA) instead of mega-doses (100-250× the RDA) might be sufficient, unless P5P-dependent enzymes are highly defective and require very high levels of P5P to function normally.

The higher level of total choline in the children with autism is interesting, and may suggest an impairment in conversion of choline to acetylcholine.

There is current interest in the role of Vitamin D in not only bone metabolism but also immune system function [47]. D3, synthesized in the skin on exposure to sunlight, and D2, supplied in plant and fungi based foods, are both prohormones for 25-OH-D which was measured in this study. The role of vitamin D in autism [48,49] has begun to receive attention. In this current study there was no difference in plasma vitamin D levels in children with autism versus controls. Measured levels for the neurotypical children were consistent with the results of the NHANES US population study. In both study groups there is a subgroup, which is borderline or below published RR, even in a geographic location (Arizona) with potential for high sun exposure. The cause for the decreased vitamin D levels in the general population is not well understood; it is likely multifactorial. One known factor is the change in modern lifestyles which have resulted in less exposure to direct sunlight. Thus, the "average" level of the general population may not be "optimal." So, it may be that most children, including children with ASD, may benefit from more vitamin D. The present results somewhat differ with a study in Egypt [19], which found that young children with autism (5.3 +/- 2.8 yr) had significantly lower levels of 25 (OH)D (28.5 +/- 16.4 mcg/l) than did controls (40.1 +/- 11.8 mcg/l) of similar age. The levels for the autism group for the Egyptian and the present study are in close agreement; however the Egyptian normative control levels are much higher than for the present study. To determine if the difference with the present results was partially due to differences in study age, the present data for children ages 5-10 years was analyzed, but found no difference between the autism and control groups. So, the difference appears to be due to differences in the control groups and may reflect differences in entire population reference ranges, perhaps due to a variety of factors, such as sun exposure, dietary intake or factors interfering in vitamin D metabolism.

It is interesting to compare the results of this study with other studies. The following findings for children with autism relative to controls are consistent with other studies: lower vitamin E [15,20]; normal levels of vitamin B12 [11,50], serum folate [11,50] and vitamin A [20]. The present finding of increased vitamin C is consistent with one study [20], but inconsistent with another study that found normal levels of vitamin C [15]. The present finding of slightly lower levels of total carotenes is in contrast to a study [15] in Saudi Arabia which found greatly increased levels of beta carotene; the reason for that difference is unclear (typically 80% of total carotene is beta-carotene, but may be due to differences in which nation the study was conducted in, due to differences in ethnicity, diet, and other factors. Fasting status is also important, as food intake rapidly affects levels of most vitamins.

### Functional tests

FIGLU is an intermediate in the deamination of histidine. Conversion of FIGLU to glutamic acid is the 4^th ^step in this process and requires the enzyme formiminotransferase and the co-factor folic acid. The elevated urinary FIGLU suggests deficiency in either the enzyme or in folic acid. Plasma folic acid was slightly lower in the autistic group but the difference was not significant. Plasma folic acid did not significantly correlate with FIGLU levels. As a water soluble vitamin, folic acid varies daily with intake. FIGLU excretion may be representative of a longer metabolic period. FIGLU may be a more sensitive indicator of need for folic acid than measurements of plasma folic acid.

N-methyl-nicotinamide is a metabolite of vitamin B3 (niacin). The finding of higher levels of urinary n-methyl-nicotinamide in the autism group (possibly significant) is consistent with another study [51] which found elevated urinary n-methyl-nicotinamide in children with autism compared to neurotypical children. Whole blood niacin levels were very similar in the autism and neurotypical groups, and whole blood niacin levels did not significantly correlate with n-methyl-nicotinamide levels, which suggests that n-methyl-nicotinamide is a more sensitive assay.

Children with autism had normal levels of kryptopyroles in urine, although a few outliers raised their standard deviation. The measurements of kryptopyroles should not be confused with measurements of the "mauve factor" which was once thought to be kryptopyrole but is actually hydroxyhemopyrrolin-2-one (HPL) [52].

### Minerals

The much lower level of WB lithium is consistent with a previous study [21], which found lower levels of lithium in younger children with autism and their mothers, but not in the older children. Those measurements were done in hair, which appears to be a more sensitive measure than blood, based on a study of lithium-deficient animals [53]. Animals fed a lithium-deficient diet had decreased immune function and suffered from more chronic infections, which may partially explain why several studies have found that children with autism have significantly more ear infections than typical children [54-57]. Lithium-deficient animals also had decreased activity of monoamine oxidase, which can greatly affect neurotransmitter levels. Low levels of lithium are associated with a wide range of psychiatric disorders [58]. The primary biochemical effect of lithium is that it regulates two enzymes, glycogen synthase kinase 3-beta and phosphatidylinositol phosphatase, both of which have been linked to autism [59,60]. Both enzymes are involved in the growth factor signaling pathway (PI3-kinase). Low levels of lithium are of concern and these results suggest that low-level lithium supplementation may be beneficial for mood stabilization in this group. Along with other studied nutrients, the role lithium plays in the etiology of autism warrants further study.

Serum iron and serum ferritin were similar in the autism and neurotypical groups. A previous study [22] found that 8 of 96 American children with ASD were anemic (haemoglobin < 110 g/l). In that study, the age range of the general group was 3-13 yr, but 7 of the 8 autism cases were in children under age 5. Another study [23] found that 16% of 96 Canadian children with ASD ages 1-10 yr had low serum ferritin (< 10-12 mcg/L), with little effect of age. The present study of older children with ASD (ages 5-16 yr) found only 2% of the children had serum ferritin levels below 12 mcg/L, which is roughly consistent with the results for older children in the study by Latif et al [22], but somewhat lower than the rate found in the study by Dosman et al 2006 [23]. Combining the results of all three studies, anemia seems to be a common problem in young children with autism (below age 5), but perhaps less common in older children with autism, likely consistent with the general population.

There was a slightly higher, but statistically significant, RBC iron in the autism group. The clinical significance of elevated RBC iron is unclear, but it did correlate with autism severity (for the PDD-BI), so elevated RBC iron may be problematic. This finding did not correlate with oxidative stress in this study; further research is warranted.

Most other minerals were only slightly different in the autism group compared to the control group. The slightly higher level of copper (WB and RBC) suggests most children with autism do not need copper supplementation. The slightly lower (possibly significant) level of magnesium in serum and WB suggests that modest magnesium supplementation might be beneficial, but RBC magnesium was normal, so at most this suggests only a minor need. The possibly significant slightly higher levels of RBC potassium, RBC phosphorus, and RBC boron seem to be minor fluctuations, and may be a statistical artifact.

The autism group had an average level of urinary iodine that was similar to the average level of the neurotypical group, but 25% of the autism group had urinary iodine levels that were below the 10^th ^percentile of the neurotypical group. 14% of the autism group and 11% of the control group had insufficient iodine (< 100 ng/ml) based on the WHO 2007 criteria, although this needs to be interpreted cautiously since those children also had lower creatinine levels (more dilute urine) and the WHO criteria is based on volume, not normalized by creatinine, since they are focused on population analyses, not individual analyses. A previous study [21] found significantly lower levels of iodine in hair of children with autism compared to controls. It is unknown if hair levels are indicative of iodine status, whereas urinary levels are accepted as the best measure of iodine status, as most iodine is excreted in the urine [61]. Overall, it does appear that there is a significant subset of children with autism with low levels of iodine, which is one of the leading causes of mental retardation worldwide [61], and we believe that further investigation of iodine status and thyroid status is warranted.

It is interesting to compare our results with a smaller study of RBC minerals in young Canadian children with autism (n = 20) compared to neurotypical children (n = 15), both with average ages of 3.9 years [24]. That study found lower levels of RBC selenium (p = 0.0006), higher levels of RBC molybdenum (p = 0.01), and possibly lower levels of RBC zinc (p = 0.08). They found similar levels of chromium, copper, magnesium, and manganese in RBC in the two groups. This smaller study partially agrees with the present study (normal levels of RBC chromium, magnesium, manganese), but partially disagrees in that the present study did not find a difference in RBC selenium, molybdenum, or zinc, and possibly found slightly higher RBC copper. Part of the difference in study results may be due to differences in ages (averages of 4 yr vs. 10 yr), geographic/dietary issues (Canada vs. Arizona), and study size.

A study [25] of copper and zinc levels of 20 British older children with autism compared to 30 British older neurotypical children did not find any significant difference in zinc or copper levels in plasma, although the autism group did have a 10% higher level of copper, similar to the present results for WB zinc (no difference) and WB copper (7% higher, p = 0.02).

A study of zinc levels in 45 children with ASD age 4-12 years compared to 41 neurotypical controls of similar age in Turkey found lower levels of zinc in WB and RBC [26]. This disagrees with the British study [25] and the present US study, possibly due to difference in culture/diet/geographical area.

### Sulfation, Methylation, Glutathione and Oxidative Stress

Sulfate is the third most abundant essential mineral in the body. Roughly 80% of sulfate is produced *in vivo *by oxidation of methionine or cysteine, both sulphur-containing amino acids which are provided from dietary proteins. Sulfation is important for many reactions including detoxification, inactivation of catecholamines, synthesis of brain tissue, sulfation of mucin proteins which line the gastrointestinal tract, and more. The measurement of total plasma sulfate involves many substances in the plasma, including neurotransmitters, steroids, glycosaminoglycans, phenols, amino acids, peptides, and other molecules. The findings of very low free and total plasma sulfate are consistent with two studies, which found decreased plasma sulfate compared to neurotypical controls [62,63]. Low sulfate in the plasma is also consistent with four studies [62,64-66] which found that children with ASD had a significantly decreased sulfation capacity compared to controls, based on low levels of the paracetamol-sulfate/paracetamol-glucuronide (PS/PG) ratio in urine following administration of paracetamol (acetaminophen, trade name of Tylenol). The finding of low plasma sulfate is also consistent with a large study that found high sulfate in the urine of children with autism [67], as sulfate wasting in the urine partly explains low levels in the plasma. ATP is required for the kidneys to resorb sulfate, and in this study ATP was correlated with levels of free and total plasma sulfate (r = 0.32 and 0.44, respectively), so this suggests that low levels of ATP are a significant contributor to decreased sulfate in children with autism. That paper also reported high levels of urinary sulfite, suggesting that there was a problem of converting sulfite to sulfate in the mitochondria. In 38% of cases (14/38) urinary sulfite and sulfate levels improved by giving 50 mcg of molybdenum, presumably since the enzyme for converting sulfite to sulfate (sulfite oxidase) contains molybdenum. In this study there was a weak correlation (r = 0.24) of RBC molybdenum with plasma free sulfate, but no significant correlation with plasma total sulfate.

The findings of decreased reduced glutathione (GSH), increased oxidized glutathione (GSSH), and increased ratio of oxidized:reduced glutathione are consistent with four previous studies [8-10,63]. The finding of decreased SAM in RBC is consistent with three studies of SAM in plasma [8-10]. Our finding of an unusually broad distribution of SAH in RBC only slightly higher (+5%, n.s.) than that of controls is similar to that of one study [10] which also found a slightly higher level of SAH in plasma (+6%, n.s.), but different from two other studies which found higher levels of SAH in plasma [8,9]. It should be noted that we measured SAM and SAH in RBC, whereas the other studies [8-10] measured SAM and SAH in plasma; RBC levels are far greater than plasma levels, but seem to provide similar results for children with autism vs. controls.

SAM is converted to SAH by the transfer of a methyl group, so the ratio of SAM/SAM is a measure of the body's methylation capacity. The finding of a slightly decreased SAM/SAH ratio (-10%, p = 0.006) is similar to one study [10] which found a slightly decreased level (-17%, n.s.), and similar to two other studies [8,9] which also found decreased levels of SAM/SAH; however, those two studies found a larger decrease (-44% and -27%, respectively) in the SAM/SAH ratio than in this study, due to lower levels of SAM.

The finding of very elevated plasma uridine suggests impaired methylation (methylation is required for conversion of uridine to thymidine), consistent with the decreased level of SAM.

The finding of elevated plasma adenosine in 33% of the autism group is consistent with two previous studies [8,9] and suggests an impairment in adenosine deaminase since adenosine levels are normal. Adenosine binds to the active site of SAH hydrolase and inactivates it [68,69] so elevated adenosine would block conversion of SAH to homocysteine, and hence may partially explain the decreased SAM/SAH ratio.

The finding of increased nitrotyrosine shows that this is also a good marker for oxidative stress in children with autism, and is consistent with the other measurements of oxidative stress (oxidized glutathione and ratio of oxidized:reduced glutathione).

Overall, the problems with SAM, glutathione, and oxidative stress suggest that children with autism need increased anti-oxidant support, folinic acid (not folic) and vitamin B12 to support the methionine cycle. (Folic acid is not sufficient in most cases. In the James et al 2004 [8] study 16 of the 20 children with autism were taking a multivitamin and mineral supplement containing 400 ug folic acid and 3 ug vitamin B-12 but still had abnormal methylation; folinic acid, not folic acid, was needed to normalize methylation).

### ATP, NADH, NADPH, CoQ10

The finding of decreased plasma ATP, NADH, and NADPH, but normal levels of their precursor (vitamin B3- niacin) may suggest an impairment in the formation of NADH from niacin. The clinical significance of decreased levels of ATP in the plasma is unclear, since most ATP is intracellular. Impaired transport of ATP throughout the body is suggested. ATP is the primary energy source for many metabolic reactions, and decreased levels of plasma ATP may be related to decreased muscle tone and decreased endurance commonly observed in children with autism [21,70] NADH is mainly involved in catabolic reactions (energy metabolism and mitochondrial function) whereas NADPH is involved in anabolic reactions (antioxidation and reductive biosynthesis) [71]. Decreased plasma ATP and NADH may also relate to impaired mitochondrial function, which has been reported in some children with autism [72-75].

The reason for the decreased ATP, NADH, and NADPH is unclear. ATP, NADH, and NADPH were all strongly correlated with one another (r = 0.67 = 0.69), and all had a significant correlation with ratio of GSSH:GSG (r = -0.34 to -0.52), and plasma nitrotyrosine (r = -0.24 to -0.51) indicating that increased oxidative stress is associated with low ATP, NADH, and NADPH. Increased oxidatative stress would result in a significant conversion of NADH to NAD+ (which was not measured in this study). This could also be true to a limited extent for NADPH, but usually NADP+ is only a very small fraction of NADPH. It also may be due to impaired function and/or amount of translocator protein in the mitochondrial membrane, which would impair transport of ATP from the mitochondria into the cytoplasm [76] and into the plasma.

### Primary Amino Acids

One significant abnormality in the plasma amino acids was elevated glutamate in the ASD group. Glutamate is the most prominent neurotransmitter. It is ubiquitous throughout the central nervous system where it modulates synaptic plasticity, vital to memory, learning and regulation and modulates gene expression, functioning in post-synaptic excitation as well as some inhibition. Overstimulation leads to excitotoxicity, creating oxidative stress, mitochondrial damage and ultimately may play a role in neurodegeneration. Peripherally it plays a role in taste, skin pain sensation and pancreatic exocrine function. Increased glutamate may be linked to some of the behavioral problems and features commonly associated with autism. It may indicate an increased need for vitamin B6, needed for conversion of glutamate to glutamine.

Another significant abnormality was decreased tryptophan in the ASD group. This may be due to decreased protein intake, and/or impaired digestion of protein into amino acids; the latter seems more likely since most of the essential amino acids were normal. Tryptophan is a precursor to the synthesis of serotonin, so decreased tryptophan is likely to impair serotonin synthesis (important in neurogenesis and neurotransmission). The importance of low tryptophan in autism was demonstrated by a randomized, double-blind, placebo-controlled crossover study that found that a 24-hour-diet low in tryptophan followed by a tryptophan-deficient amino acid drink led to a significant worsening in behavior in adults with autism [77].

There were several other differences which were only possibly significant (p < 0.05). The ASD group also had slightly increased serine, and slightly decreased phenylalanine and tyrosine. The low tyrosine is presumably due to the low phenylalanine, since tyrosine is derived from phenylalanine, and they were highly correlated with one another (r = 0.74). The low phenylalanine could be due to decreased protein intake and/or impaired digestion of protein into those amino acids.

Our results suggest that some children with autism (those with low tryptophan and phenylalanine) would benefit from either increased protein intake, use of digestive enzymes containing proteases, and/or supplements of tryptophan (or 5-HTP) and phenylalanine.

Two previous studies by James et al [8,9] found decreased plasma methionine in children with autism vs. controls, but their most recent study [10] did not; the latter is consistent with the present study, which did not find a significant difference in plasma methionine.

Although there have been several other studies of plasma amino acids in children with autism, they suffered from one or more limitations, including small sample size, no fasting requirement, lack of neurotypical controls, and/or age differences between the autism and control groups, and thus have not surprisingly yielded contradictory results to one another, and to the present results.

Overall, the present data on plasma amino acids appears to be more reliable than most of the other studies of plasma amino acids, due to its larger size, overnight fast, use of neurotypical controls, use of controls of similar age, and simultaneous testing of autism and control groups. The present results suggest that children with autism, on average, have lower levels of plasma tryptophan, increased plasma glutamate, and possibly lower levels of several other amino acids.

### Secondary Amino Acids

The only significant abnormality in the secondary amino acids was increased beta-aminoisobutyrate (also known as 3-aminoisobutyric acid) in the autism group. Beta-aminoisobutyrate is a product formed by the catabolism of thymine, one of the four nucleobases in the nucleic acid of DNA. So, elevated beta-aminoisobutyrate may indicate an increased rate of DNA turnover, and/or it may indicate an inhibition of the conversion of beta-aminoisobutyrate into the intermediates that eventually lead to the citric acid cycle.

There was also a possibly significant lower level of taurine, which could be due to an impairment of the conversion of methionine to cysteine to taurine, but it could also be due in part to increased wasting of taurine in the urine, which has also been reported [78]. This is consistent with another study [63], which also found low plasma taurine in children with autism, although that study found a greater difference.

For "homocystine + homocysteine", it was undetectable in most cases, but it was detectable in a higher fraction of the autism group (29%) vs. the control group (11%), suggesting a possible abnormality in metabolism of homocystine and/or homocysteine in some children, but due to measurement limitations this requires investigation with more sensitive techniques that do not oxidize the homocysteine.

### Potential Confounding Factors: Medication, Gastrointestinal and Dietary Issues

45% of the autism group were taking medications, and some of those can affect nutritional status, metabolism, and gastrointestinal function. However, a t-test comparison of the two groups revealed only two small differences between the autism groups taking and not taking medications, so medication use appeared to have little effect on the results reported here. Many of the autism group had gastrointestinal problems, either separate from or secondary to medications being taken, which may have affected nutritional intake. As shown in Table 1, 18% of the autism group had moderate or severe diarrhea, and 41% had moderate or severe constipation (9% of the total participants had both moderate or severe diarrhea and constipation, apparently alternating between them). Also, 53% of the children had moderate or severe problems with self-restricted diets (only interested in eating a few foods, often resulting in poor nutritional intake). Finally, 16% of the children with autism were on special diets (see Table 1), which could result in some differences in nutritional status.

### Correlation Analysis

In most cases the sign of the correlation analysis is consistent with our understanding. For example, low amounts of an essential mineral (sulfate) or essential amino acid (phenylalanine) correlated with more severe autism. The positive correlation of RBC iron with autism severity suggests that excess RBC iron requires further research. Excess iron could increase oxidative stress through the Fenton reaction, in which iron catalyzes the conversion of hydrogen peroxide into two hydroxyl radicals and water.

### Regression Analysis

It is very interesting that almost all of the biomarker groups have significant associations with variation in autism severity. Vitamins (especially vitamin B6, vitamin C, and n-methyl-nicotinamide) had a very high adjusted R^2 ^for the PDD-BI (0.57) (see Table 13). Minerals (especially calcium, iron, and zinc) had moderate adjusted R^2 ^(0.22-0.38), as did the primary amino acids (adjusted R^2 ^of 0.22-0.38) (see Table 13). Secondary amino acids and the group of Sulfation/Methylation/Glutathione/Oxidative Stress had significant but only modest adjusted R^2 ^(0.15-0.26) (see Table 14 and 15). The group of ATP/NADH/NADPH/CoQ10 had a significant association for only one of the three severity measures (see Table 14). So, overall, it seems that certain vitamins, minerals, amino acids, and (to a lesser extent) other biomarkers have significant associations with variations in the severity of autism (see Table 16).

**Table 13:** Regression Analysis for Autism Severity vs. Vitamins (and related metabolites) and Minerals

	PDD-BI	ATEC	SAS
**Vitamins**			

Adjusted R^2^	0.57	0.25	0.26

P-value	0.0008	0.005	0.02

Primary variables	Vit B6*** Vit C*** N-Methy-nicotinamide*** Vit K** Vit B2* Lipoic Acid * Kryptopyroles*	Vit C ** N-methyl-nicotinamide *	Vit C *** Vit B6 ** N-methyl-nicotinamide ** Vit K *

**Minerals**			

Adjusted R^2^	0.38	0.22	0.27

P-value	0.0007	0.007	0.003

Primary variables	Calcium (RBC) *** Iron (RBC) ** Zinc (WB) ** Selenium (WB) ** Manganese (RBC) * Copper (WB) * Lithium (WB) * Zinc (RBC) *	Iron (RBC) *** Calcium (RBC) ** Potassium (RBC) * Zinc (WB) *	Iron (RBC) *** Calcium (RBC) ** Zinc (RBC) ** Potassium (RBC) ** Vanadium (RBC) *

* variable has a significance of p < 0.05

** variable has a significance of p < 0.01

*** variable has a significance of p < 0.001

**Table 14:** Regression analysis of autism severity and plasma amino acids

	PDD-BI	ATEC	SAS
**Primary Plasma Amino Acids (20)**			

Adjusted R^2^	0.39	0.22	0.24

P-value	0.001	0.01	0.007

Primary variables	Proline ** Serine ** Leucine** Valine * Taurine *	Asparagine ** Tryptophan * Glutamic Acid * Glutamine * Phenylalanine *	Serine ** Asparagine * Proline *

**Other Amino Acids**			

Adjusted R^2^	0.23	0.26	0.18

P-value	0.008	0.005	0.04

Primary variables	Beta-amino-isobutyrate ** Ethanolamine * Sarcosine *	Ethanolamine ** Sarcosine ** Beta-amino-isobutyrate ** 1-methyl-histidine *	Ethanolamine *

* variable has a significance of p < 0.05

** variable has a significance of p < 0.01

*** variable has a significance of p < 0.001

**Table 15:** Regression Analysis for Autism Severity vs. Sulfation/Methylation/Glutathione/Oxidative Stress, and vs. ATP/NADH/NADPH/CoQ10

	PDD-BI	ATEC	SAS
**Sulfation, Methylation, Glutathione, Oxidative Stress**			

Adjusted R^2^	0.20	0.15	0.24

P-value	0.005	0.03	0.002

Primary variables	Free Sulfate ** SAM * Oxidized Glut. *	Free Sulfate ** SAM * Oxidized Glut. *	Free Sulfate ** Oxidized Glutat. * Adenosine *

**ATP, NADH, NADPH, CoQ10**			

Adjusted R^2^		0.13	

P-value	n.s.	0.01	n.s.

Primary variables		NADH ** ATP *	

* variable has a significance of p < 0.05

** variable has a significance of p < 0.01

*** variable has a significance of p < 0.001

**Table 16:** Regression analysis of autism severity vs. highly significant biomarkers (defined as p < 0.01, in a previous regression analysis)

	PDD-BI	ATEC	SAS
**Significant Biomarkers**			

Adjusted R^2^	0.56	0.24	0.42

P-value	0.000003	0.002	0.0002

Primary variables	Iron (RBC) *** Methylmalonic Acid ** Glutamate ** Proline ** Serine	Sarcosine * Ethanolamine * Beta-amino-isobutyrate * Free Sulfate *	Potassium (RBC) ** Calcium (RBC) ** N-Methyl-nicotinamide ** Serine ** Phenylalanine ** Alanine *

* variable has a significance of p < 0.05

** variable has a significance of p < 0.01

*** variable has a significance of p < 0.001

The Overall analysis yielded very significant associations with the variation in autism severity, especially for the PDD-BI, followed by the SAS and then the ATEC. The most significant biomarkers were generally different for each autism severity instrument, but this is not surprising since some biomarkers have significant correlations with one another, and small differences can lead to one biomarker replacing another in the analysis. Rather than focusing on a particular biomarker, the Overall analysis is best interpreted as demonstrating that variations in the severity of autism are strongly associated with the nutritional and metabolic biomarkers investigated in this study. Of course, other factors not addressed in this study also contribute to the severity of autism, and all of these factors are worthy of further research.

These significant associations may offer clues to the etiology of autism. Vitamins, minerals, and essential amino acids are, by definition, essential for human life, so low levels of those critical nutrients will impair metabolic pathways and may contribute to the developmental delays which occur in autism. We hypothesize that the nutritional and metabolic problems reported in this study may be associated with the etiology of autism. We also hypothesize that correction of these problems may help reduce some of the symptoms and co-morbidities of autism, although much more research is needed to investigate these hypotheses.

### Comparison with other studies

There are two other general studies that are also interesting to compare with our results. One study [18] found inadequate intake of folic acid, vitamin B6, calcium, vitamin A, vitamin C, and zinc in most children with autism in China. However, they only assessed dietary intake, and compared dietary intake based on established literature values (no control group); they did not measure blood levels.

It is also interesting to compare our results with a study by Mehl-Madrona et al 2010 [79]. The present results suggest that micronutrient therapy with vitamins and minerals would be beneficial, and that is consistent with their study, which found that micronutrient supplementation was comparable with pharmaceuticals in terms of improvements in the Childhood Autism Rating Scale, and resulted in better improvements on the Aberrant Behavior Checklist (p < 0.0001), Clinical Global Impressions (p = 0.003), and Self-Injurious Behavior (p = 0.005). Similarly, a small randomized, double-blind, placebo-controlled study of a multi-vitamin/mineral supplement found that it was beneficial to children with autism, primarily in reducing gastrointestinal problems and sleep problems [45].

## Limitations of this study

1) The diagnosis of an autism spectrum disorder by a qualified medical professional was verified in writing, but there no additional verification. Similarly, for the neurotypical children, no additional verification was made beyond the parental report.

2) The sample size of 55 children with ASD and 44 neurotypical children was large enough to observe many significant differences between the two groups, but some differences were only marginally or possibly significant - another study, preferably with larger number of participants, is needed to verify some of the observations.

3) Although several functional tests of need for vitamins were conducted, several more could have been added, but there were limitations on the total amount of tests that could be done with the blood that was drawn.

4) Medication effects: 45% of the children with autism were taking one or more medications (see Table 1), but those medications appear to have had little effect on the study results (see Medication Effects in the Results section).

5) Dietary effects: 16% of the children with autism were on special diets (see Table 1), which might have had some effect on the results.

6) All the study participants were from Arizona, so the results for this region may be somewhat different from other parts of the US or the world.

## Conclusions

Many significant differences (p < 0.001) were observed in the autism group compared to the neurotypical group, including low levels of biotin, plasma glutathione, RBC SAM, plasma uridine, plasma ATP, RBC NADH, RBC NADPH, plasma sulfate (free and total), and plasma tryptophan; and high levels of oxidative stress markers and plasma glutamate. Decreased ATP may be a contributing factor to decreased sulfate and decreased SAM/methylation. Decreased NADPH may be a cause of the increased oxidation of glutathione. This study replicated previous findings of very low lithium in children with ASD, but the p-value was only 0.006 (possibly significant). There were also many marginal differences, and many possible differences, but a larger study is needed to confirm the validity of those observations. Overall, it appears that children with autism do have many abnormalities in their nutritional and metabolic status. The underlying causal relationships of these abnormalities are not yet well understood. An important issue in the clinical care of ASD children is that most vitamins, minerals, and plasma amino acids were within the reference range, but other biomarkers (oxidative stress, methylation, sulfation) were very abnormal, suggesting that those other biomarkers can be important guides for treatment.

The regression analysis found that some vitamins, minerals, amino acids, and (to a lesser extent) other biomarkers are significantly associated with variations in the severity of autism, with vitamins being especially important.

We hypothesize that support for these nutritional and metabolic problems by increasing nutrient intake may reduce the symptoms and co-morbidities that are associated with autism. These nutritional and metabolic dysfunctions may be related to the etiology of autism. Certainly much more research is needed to investigate these hypotheses.

## Competing interests

The authors declare that they have no competing interests.

## Authors' contributions

JBA was the principal investigator, oversaw the study design, conducted most of the data analysis, and wrote most of the paper. TA oversaw the laboratory measurements at Health Diagnostics, and assisted with interpreting the results and editing the paper. SMM was the study physician, oversaw patient care, and assisted with interpreting the results and editing the paper. RAR assisted with statistical analysis and editing the paper. DQ oversaw the laboratory measurements at Doctors Data, and assisted with interpreting the results and editing the paper. EG was the lead study nurse, and supervised many of the study participants. EG was the study coordinator, and assisted with participant recruitment and study oversight. ML, JM, SA, and SB were study nurses and supervised study participants. WL assisted with data entry and analysis. All authors read and approved the final version of the paper.
